# Supplementary material for: Factors associated with SARS-CoV-2 infection in unvaccinated children and young adults
Source: BMC Infect Dis. 2024 Jan 15;24:91. doi: 10.1186/s12879-023-08950-1 (PMC10790408; doi:10.1186/s12879-023-08950-1)
Supplement: Supplementary file 2 — Supplementary Material 2: Supplementary Fig. 1: Timeline of COVID-19 pandemic and provincial milestones through June 2022 by SARS-CoV-2 variant of concern. [file 12879_2023_8950_MOESM2_ESM.docx]

**Supplementary Figure 1: Timeline of COVID-19 pandemic and provincial milestones through June 2022 by SARS-CoV-2 variant of concern^1^**


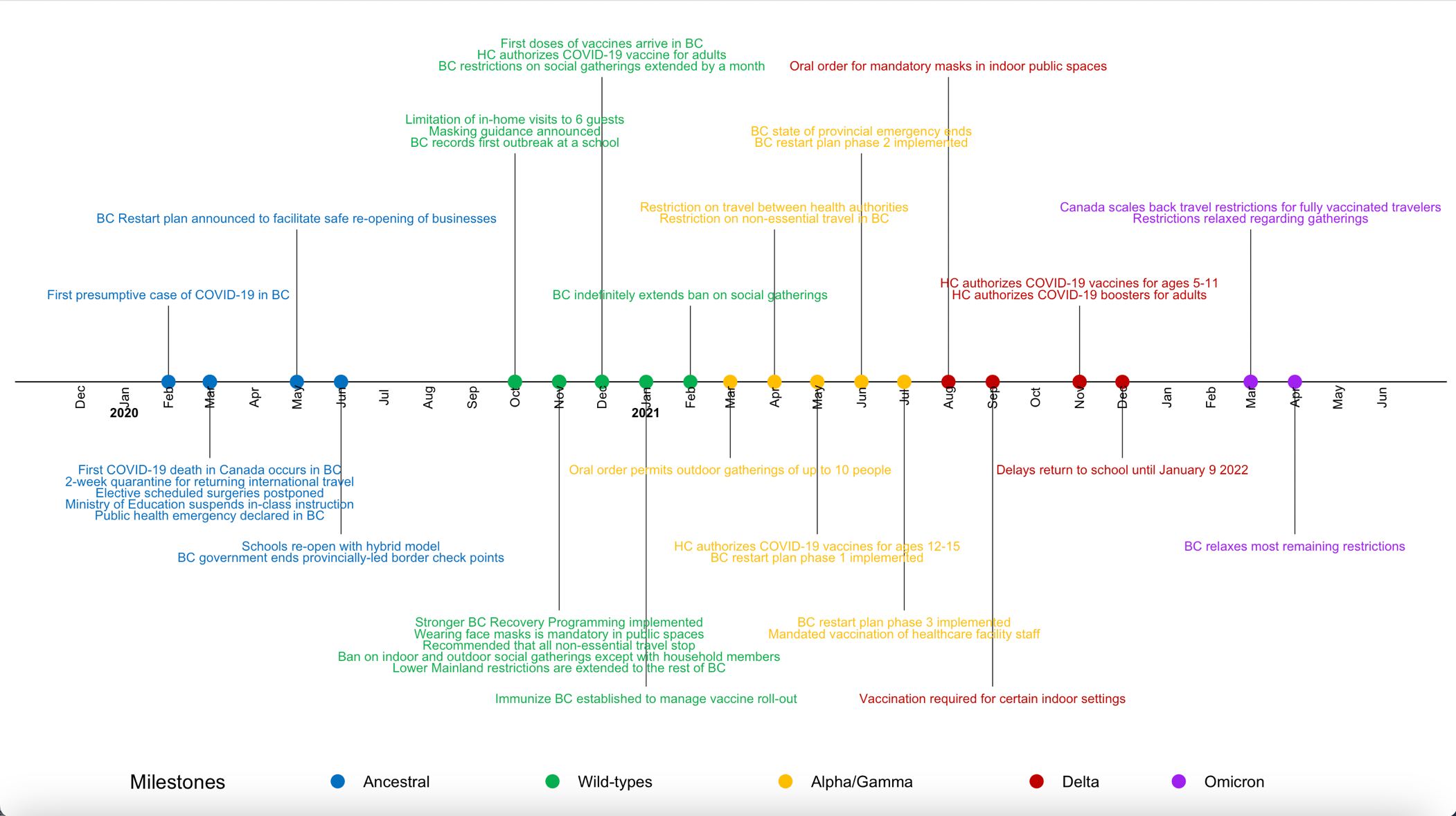


^1^BC refers to British Columbia; HC refers to Health Canada
