# Supplementary material for: Factors associated with SARS-CoV-2 infection in unvaccinated children and young adults
Source: BMC Infect Dis. 2024 Jan 15;24:91. doi: 10.1186/s12879-023-08950-1 (PMC10790408; doi:10.1186/s12879-023-08950-1)
Supplement: Supplementary file 3 — Supplementary Material 3: Supplementary Fig. 2: Case report form. [file 12879_2023_8950_MOESM3_ESM.pdf]

Baseline Survey

Record ID:

BACKGROUND INFORMATION

Please answer the following questions about yourself.

PARENTS: if you are answering this survey on behalf of your child, please answer the questions in relation to your child.

What is your (your child's) age? ____ ○ Years ○ Months

You (your child) must be under 25 years old to participate in this study. If you (your child) are 25 years old or older, please contact the SPRING study team.
If you are entering a child's age, please enter in months if under 2 years of age.

What sex were you (your child) assigned at birth?
* must provide value

- ○ Male
- ○ Female
- ○ Intersex
- ○ Prefer to self-describe as: ____
- ○ Prefer not to answer

What best describes your (your child's) gender:
* must provide value

- ○ Man/boy
- ○ Woman/girl
- ○ Non-binary, GenderQueer, Agender or similar identity
- ○ Two-spirit
- ○ Prefer to self-describe as: ____
- ○ Prefer not to answer

What are the first three digits of your postal code? (e.g. V5H) * must provide value ____

What best describes your (your child's) ethnicity? *(tick all that apply)*
* must provide value

- ☐ Indigenous (First Nations, Metis, Inuk/Inuit)
- ☐ White
- ☐ Chinese
- ☐ South Asian (East Indian, Pakistani, Sri Lankan, etc)
- ☐ Black (African, African-American)
- ☐ Filipino
- ☐ Latin, Central and South American
- ☐ Southeast Asian (Vietnamese, Cambodian, Malaysian, Laotian, etc.)
- ☐ Arabic
- ☐ West Asian (Iranian, Afghan, etc.)
- ☐ Korean
- ☐ Japanese
- ☐ Alaskan Native
- ☐ Native Hawaiian or other Pacific Islander
- ☐ Other, specify: ____
- ☐ Prefer not to answer

Do you (your child) have any current medical conditions?
* must provide value

- ○ Yes
- ○ No

reset

Please list your (your child's) current medical condition(s): ____

Are you (your child) currently taking any prescription medication?
* must provide value

- ○ Yes
- ○ No
- ○ Prefer not to answer

reset

Please list the name of the prescription medication(s): ____

Baseline Survey

Since January 2020, did you (your child) leave the home to go to:
* must provide value

- ☐ Work *(choose all that apply)*
  - ☐ Healthcare worker
  - ☐ Essential worker (e.g. postal, grocery, delivery)
  - ☐ Personal service provider (e.g. hairdresser/barber, esthetician)
  - ☐ Teacher, Principal, School counselor etc.
  - ☐ Other, specify:
- ☐ School *(choose level of schooling)*
  - ○ Preschool
  - ○ Elementary/Primary
  - ○ Secondary/High school
  - ○ Post secondary (college, university or technical institute)
- ☐ Daycare
- ☐ Other, specify: ____
- ☐ None of the above
- ☐ Prefer not to answer

In the last 6 months, on average, how many times per week do you (your child) use the following transportation?

	never	less than 1/week	1-2 times/week	3-4 times/week	5-6 times/week	daily
**Public transportation** * must provide value	○	○	○	○	○	○ reset
**Another's private vehicle (such as taxi, car pooling, ride share)** * must provide value	○	○	○	○	○	○ reset

COVID-19 EXPOSURE INFORMATION

Since January 2020, have you (your child) travelled outside of BC?
(tick all that apply)
* must provide value

- ☐ Yes, within Canada
- ☐ Yes, outside Canada
- ☐ No
- ☐ Prefer not to answer

Which province did you (your child) travel to?
* must provide value

- ☐ Alberta
- ☐ Manitoba
- ☐ New Brunswick
- ☐ Newfoundland and Labrador
- ☐ Northwest Territories
- ☐ Nova Scotia
- ☐ Nunavut
- ☐ Ontario
- ☐ Prince Edward Island
- ☐ Quebec
- ☐ Saskatchewan
- ☐ Yukon
- ☐ Prefer not to answer

How many total weeks did you (your child) spend outside of BC but within Canada? ____
* must provide value

When did you (your child) last return to BC from within Canada? ____ Today D-M-Y
* must provide value

What country/countries did you (your child) travel to? ____
* must provide value

How many total weeks did you (your child) spend outside of Canada? ____
* must provide value

When did you (your child) last return to BC from outside Canada? ____ Today D-M-Y
* must provide value

Baseline Survey

Have you (your child) ever had any exposure to a person who tested positive for COVID-19?
* must provide value

- ◯ Yes
- ◯ No
- ◯ I don't know
- ◯ Prefer not to answe

reset

How many people have you (your child) been exposed to?
* must provide value

______

Exposure #1

Please identify where the exposure occurred: (check all that apply)

- ☐ Household member
- ☐ Non-household close contact (friend/other family)
- ☐ Health care setting (e.g. Dr. office, physiotherapis/chiropractor office, hospital)
- ☐ School, college, university
- ☐ Personal service provider (e.g. hair salon/barber, esthetician)
- ☐ Workplace
- ☐ Travel, where:
- ☐ Other, specify: ______
- ☐ Prefer not to answer

When did this exposure occur?

Month: ______ **Year:** ______

At the time of exposure, do you think the person was still infectious (this is typically within the first 14 days of their illness)?

- ◯ Yes
- ◯ No
- ◯ I don't know
- ◯ Prefer not to answer

Exposure #2

Please identify where the exposure occurred: (check all that apply)

- ☐ Household member
- ☐ Non-household close contact (friend/other family)
- ☐ Health care setting (e.g. Dr. office, physiotherapis/chiropractor office, hospital)
- ☐ School, college, university
- ☐ Personal service provider (e.g. hair salon/barber, esthetician)
- ☐ Workplace
- ☐ Travel, where:
- ☐ Other, specify: ______
- ☐ Prefer not to answer

When did this exposure occur?

Month: ______ **Year:** ______

At the time of exposure, do you think the person was still infectious (this is typically within the first 14 days of their illness)?

- ◯ Yes
- ◯ No
- ◯ I don't know
- ◯ Prefer not to answer

Exposure #3

Please identify where the exposure occurred: (check all that apply)

- ☐ Household member
- ☐ Non-household close contact (friend/other family)
- ☐ Health care setting (e.g. Dr. office, physiotherapis/chiropractor office, hospital)
- ☐ School, college, university
- ☐ Personal service provider (e.g. hair salon/barber, esthetician)
- ☐ Workplace
- ☐ Travel, where:
- ☐ Other, specify: ______
- ☐ Prefer not to answer

When did this exposure occur?

Month: ______ **Year:** ______

At the time of exposure, do you think the person was still infectious (this is typically within the first 14 days of their illness)?

- ◯ Yes
- ◯ No
- ◯ I don't know
- ◯ Prefer not to answer

Are you (your child) currently in isolation?

- ◯ Yes
- ◯ No

reset

When you (your child) were in indoor public spaces (e.g. shopping, dining) how frequently did you (your child):

	Never	Rarely	Some of the time	Most of the time	Every time
**Wash your hands or use hand sanitizer** * must provide value	◯	◯	◯	◯	◯
**Maintain a distance of 2 meters from members of the public** * must provide value	◯	◯	◯	◯	◯
**Wear a mask or other face covering** * must provide value	◯	◯	◯	◯	◯

reset

reset

Baseline Survey

HOUSEHOLD DETAILS

Other than yourself (your child), how many people live in your home since January 2020?
If you (your child) are a student currently living in student residence, please complete the following table for each roommate you have.

* must provide value

Please complete the following table(s) for each household member:

Household member #1

**Age in years** (enter as "0" for children less than 1yr old)	
**In the last 6 months, did this household member leave the home to go to:** (tick all that apply)	☐ Work *(choose all jobs in the last 6 months)*<br>☐ Healthcare worker<br>☐ Essential worker (e.g. postal, grocery, delivery)<br>☐ Personal service provider (e.g. hairdresser/barber, esthetician)<br>☐ Teacher, Principal, School counsellor etc.<br>☐ Other, specify:<br>☐ School *(choose level of schooling)*<br>○ Preschool<br>○ Elementary/Primary<br>○ Secondary/High school<br>○ Post secondary (college, university or technical institute)<br>reset<br>☐ Daycare<br>☐ Other, specify:<br>☐ No, none of the above

Household member #2

**Age in years** (enter as "0" for children less than 1yr old)	
**In the last 6 months, did this household member leave the home to go to:** (tick all that apply)	☐ Work *(choose all jobs in the last 6 months)*<br>☐ School *(choose level of schooling)*<br>☐ Daycare<br>☐ Other, specify:<br>☐ No, none of the above

⋮

Household member #9

**Age in years** (enter as "0" for children less than 1yr old)	
**In the last 6 months, did this household member leave the home to go to:** (tick all that apply)	☐ Work *(choose all jobs in the last 6 months)*<br>☐ School *(choose level of schooling)*<br>☐ Daycare<br>☐ Other, specify:<br>☐ No, none of the above

Baseline Survey

Since January 2020, have you (your child) been sick?
* must provide value

- ○ Yes
- ○ No
- ○ Prefer not to answer

reset

How many times have you (your child) been sick?
* must provide value

[ ]

Illness #1	Illness #2	Illness #3
**When was this illness? (Month/year)**		
[ ] [ ]	[ ] [ ]	[ ] [ ]
**Which of the following symptoms did you (your child) experience during this illness? (tick all that apply)**		
☐ Fever >38 C<br>☐ Chills<br>☐ Cough<br>☐ Shortness of breath<br>☐ Alteration to taste<br>☐ Alteration to smell<br>☐ Nausea<br>☐ Vomiting<br>☐ Diarrhea<br>☐ None of the above<br>☐ Prefer not to answer	☐ Fever >38?C<br>☐ Chills<br>☐ Cough<br>☐ Shortness of breath<br>☐ Alteration to taste<br>☐ Alteration to smell<br>☐ Nausea<br>☐ Vomiting<br>☐ Diarrhea<br>☐ None of the above<br>☐ Prefer not to answer	☐ Fever >38?C<br>☐ Chills<br>☐ Cough<br>☐ Shortness of breath<br>☐ Alteration to taste<br>☐ Alteration to smell<br>☐ Nausea<br>☐ Vomiting<br>☐ Diarrhea<br>☐ None of the above<br>☐ Prefer not to answer
**How long did this illness last?**		
[ ]<br>○ Days<br>○ Weeks<br>reset	[ ]<br>○ Days<br>○ Weeks<br>reset	[ ]<br>○ Days<br>○ Weeks<br>reset
**Did this illness cause you (your child) to miss work or school/daycare?**		
○ Yes<br>○ No<br>reset	○ Yes<br>○ No<br>reset	○ Yes<br>○ No<br>reset
**Did you (your child) need to seek medical attention?**		
○ Yes<br>○ No<br>reset	○ Yes<br>○ No<br>reset	○ Yes<br>○ No<br>reset

You mentioned above that you sought medical attention, did you (your child) require hospitalization?
* must provide value

- ○ Yes
- ○ No
- ○ Prefer not to answer

reset

Did you (your child) require admission to ICU?
* must provide value

- ○ Yes
- ○ No
- ○ Prefer not to answer

reset

Are you (your child) still sick?
* must provide value

- ○ Yes
- ○ No
- ○ Prefer not to answer

reset

Have you (your child) ever been tested for COVID-19?
* must provide value

- ○ Yes
- ○ No
- ○ Prefer not to answer

reset

When were you (your child) last tested?
* must provide value

mo: [ ] yr: [ ]

What was the result?
* must provide value

- ○ Positive
- ○ Negative
- ○ Indeterminate/Inconclusive
- ○ I don't know
- ○ Prefer not to answer

reset

Baseline Survey

Have you (your child) been vaccinated against COVID-19?
* must provide value

- ○ Yes
- ○ No

reset

How many doses of the COVID-19 vaccine have you (your child) received so far?
* must provide value

- ○ One dose
- ○ Two dose
- ○ More than two dose

Vaccination date of first dose:

Day	Month	Year

* must provide value

Which vaccine did you (your child) receive?
* must provide value

- ○ Pfizer and BioNTech mRNA vaccine
- ○ Moderna mRNA vaccine
- ○ AstraZeneca Oxford vaccine
- ○ Janssen (Johnson & Johnson) vaccine
- ○ Medicago vaccine
- ○ Novavax vaccine
- ○ Sanofi/GSK vaccine
- ○ Other: ______
- ○ Don't know

Vaccination date of second dose:

Day	Month	Year

* must provide value

Which vaccine did you (your child) receive?
* must provide value

- ○ Pfizer and BioNTech mRNA vaccine
- ○ Moderna mRNA vaccine
- ○ AstraZeneca Oxford vaccine
- ○ Janssen (Johnson & Johnson) vaccine
- ○ Medicago vaccine
- ○ Novavax vaccine
- ○ Sanofi/GSK vaccine
- ○ Other: ______
- ○ Don't know

Vaccination date of additional dose:

Day	Month	Year

* must provide value

Which vaccine did you (your child) receive?
* must provide value

- ○ Pfizer and BioNTech mRNA vaccine
- ○ Moderna mRNA vaccine
- ○ AstraZeneca Oxford vaccine
- ○ Janssen (Johnson & Johnson) vaccine
- ○ Medicago vaccine
- ○ Novavax vaccine
- ○ Sanofi/GSK vaccine
- ○ Other: ______
- ○ Don't know

Next we'd like to ask you some questions about how you feel about vaccines, including a future COVID-19 vaccine:
For parents of children < 14 years old, please answer the following questions for yourself:

	Very unlikely	Unlikely	Neutral	Somewhat likely	Very likely
If a COVID-19 vaccine were to become available to the public, and recommended for you, how likely are you to receive it?<br>* must provide value	○	○	○	○	○

Baseline Survey

Question	Options
Do you receive all recommended vaccines when offered to you by health care professionals?	◯ Yes<br>◯ No<br>◯ I don't know
Thinking back to before the beginning of the pandemic (December 2019), how much did you value vaccines?	◯ No value<br>◯ Very little value<br>◯ Neutral<br>◯ A little value<br>◯ Valued a lot
Today, how much do you value vaccines?	◯ No value<br>◯ Very little value<br>◯ Neutral<br>◯ A little value<br>◯ Valued a lot
Have you received the influenza vaccine in the past 5 years?	◯ Never<br>◯ 1-2 times<br>◯ 3-4 times<br>◯ Every year<br>◯ I don't know

Variable: kab_next_fluvaccine

Question	Options
Do you intend to or did you receive the influenza vaccine in the fall of 2020?	◯ Yes<br>◯ No<br>◯ I don't know

@HIDDEN

Variable: kab_last_fluvaccine

Question	Options
Did you receive the influenza vaccine in the fall of [last year]?	◯ Yes<br>◯ No<br>◯ I don't know

Variable: kab_current_fluvaccine

Question	Options
Have you received the influenza vaccine in the fall of [current year]?	◯ Yes<br>◯ No<br>◯ I don't know

Variable: kab_intend_fluvaccine *Branching logic:* [kab_current_fluvaccine] = 'N'

Question	Options
Do you intend to receive the influenza vaccine in [current year]?	◯ Yes<br>◯ No<br>◯ I don't know

Baseline Survey

Please indicate how much you agree or disagree with the following statements:

	Strongly disagree	Somewhat disagree	Neutral	Somewhat agree	Strongly agree
Childhood vaccines are important for a child's health	○	○	○	○	○
Getting vaccines is a good way to protect children from disease	○	○	○	○	○
Childhood vaccines are effective	○	○	○	○	○
Having a child vaccinated is important for the health of others in my community	○	○	○	○	○
All childhood vaccines offered by the BC immunization program in my community are beneficial	○	○	○	○	○
The information I receive about vaccines from the vaccination program is reliable and trustworthy	○	○	○	○	○
Generally, I do what my doctor or health care provider recommends about vaccines	○	○	○	○	○
New vaccines carry more risks than older vaccines	○	○	○	○	○
I am concerned about potential serious adverse effects of vaccines	○	○	○	○	○
Children do not need vaccines for diseases that are not common anymore	○	○	○	○	○
The risk associated with diseases is larger than the risk associated with vaccinations	○	○	○	○	○

Baseline Survey

Assuming that a safe and effective COVID-19 vaccine was available and recommended, please indicate how much you agree or disagree with the following statements:

	Strongly disagree	Disagree	Neutral	Agree	Strongly agree
A COVID-19 vaccine would be beneficial<br>* must provide value	○	○	○	○	○
A COVID-19 vaccine would be beneficial for children<br>* must provide value	○	○	○	○	○
A COVID-19 vaccine would be beneficial for individuals 60-years and older<br>* must provide value	○	○	○	○	○
COVID-19 is a serious illness<br>* must provide value	○	○	○	○	○
A COVID-19 vaccine would be beneficial for the health of my community<br>* must provide value	○	○	○	○	○
A COVID-19 vaccine would be safe<br>* must provide value	○	○	○	○	○
A COVID-19 vaccine would be effective in preventing COVID-19<br>* must provide value	○	○	○	○	○
A COVID-19 vaccine should be mandatory<br>* must provide value	○	○	○	○	○

Please answer the following questions, with the assumption that a safe and effective COVID-19 vaccine is available and recommended.

	Strongly disagree	Disagree	Neutral	Agree	Strongly agree
Most people who are important to me would think that I should receive the COVID-19 vaccine	○	○	○	○	○
People who are important to me would expect me to receive the COVID-19 vaccine	○	○	○	○	○
I would feel under social pressure to receive a COVID-19 vaccine	○	○	○	○	○
Everyone I know would get the COVID-19 vaccine	○	○	○	○	○
My family physician's (or other primary Health Care Provider's) opinion is important to me	○	○	○	○	○
What the BC Public Health Officer recommends is important to follow	○	○	○	○	○
What my coworkers think is important to me	○	○	○	○	○
What my employer/work institution thinks is important to me	○	○	○	○	○
What my school/children's school thinks is important to me	○	○	○	○	○
What my friends think is important to me	○	○	○	○	○
What my family thinks is important to me	○	○	○	○	○

Baseline Survey

Please answer the following questions, with the assumption that a safe and effective COVID-19 vaccine is available and recommended.

	Strongly disapprove	Disapprove	Neutral	Approve	Strongly approve
My family physician (or other primary Health Care Provider) would [approve/disapprove] of me receiving a COVID-19 vaccine	○	○	○	○	○
The BC Public Health Officer would [approve/disapprove] of me receiving the COVID-19 vaccine	○	○	○	○	○
My coworkers would [approve/disapprove] of me receiving the COVID-19 vaccine	○	○	○	○	○
My employer/work institution would [approve/disapprove] of me receiving the COVID-19 vaccine	○	○	○	○	○
The educational institution (elementary/high school/college/university) that I or my children attend/are associated with would [approve/disapprove] of me receiving the COVID-19 vaccine	○	○	○	○	○
My friends would [approve/disapprove] of me receiving the COVID-19 vaccine	○	○	○	○	○
My family would [approve/disapprove] of me receiving the COVID-19 vaccine	○	○	○	○	○

For the next 3 questions: If a COVID-19 vaccine was publicly funded and available, like the flu shot, ...

	Strongly disagree	Disagree	Neutral	Agree	Strongly agree
...It would be difficult to receive the COVID-19 vaccine	○	○	○	○	○
... I could easily receive a COVID-19 vaccine if I wanted to	○	○	○	○	○
... It would be completely up to me whether I received the COVID-19 vaccine	○	○	○	○	○

How much control do you feel you would have over whether you receive a COVID-19 vaccine?

- ○ Very little control
- ○ Not much control
- ○ Neutral
- ○ Some control
- ○ A lot of control

Please make sure you click the "submit" button to complete your survey. Your survey responses will not be registered if you do not click "submit".

Baseline Survey-household

Record ID:

BACKGROUND INFORMATION

PARENTS: Please answer the questions in relation to your child

What is your child's age? [ ] ◯ Years ◯ Months

Your child must be under 25 years old to participate in this study. If your child is 25 years old or older, please contact the SPRING study team.
If your child is under 2 years of age, please enter the age in months.

What sex was your child assigned at birth?
* must provide value

- ◯ Male
- ◯ Female
- ◯ Intersex
- ◯ Prefer to self-describe as: [ ]
- ◯ Prefer not to answer

What best describes your child's gender:
* must provide value

- ◯ Man/boy
- ◯ Woman/girl
- ◯ Non-binary, GenderQueer, Agender or similar identity
- ◯ Two-spirit
- ◯ Prefer to self-describe as: [ ]
- ◯ Prefer not to answer

What are the first three digits of your child's postal code? (e.g. V5H) * must provide value [ ]

What best describes your child's ethnicity? ***(tick all that apply)***
* must provide value

- ☐ Indigenous (First Nations, Metis, Inuk/Inuit)
- ☐ White
- ☐ Chinese
- ☐ South Asian (East Indian, Pakistani, Sri Lankan, etc)
- ☐ Black (African, African-American)
- ☐ Filipino
- ☐ Latin, Central and South American
- ☐ Southeast Asian (Vietnamese, Cambodian, Malaysian, Laotian, etc.)
- ☐ Arabic
- ☐ West Asian (Iranian, Afghan, etc.)
- ☐ Korean
- ☐ Japanese
- ☐ Alaskan Native
- ☐ Native Hawaiian or other Pacific Islander
- ☐ Other, specify: [ ]
- ☐ Prefer not to answer

Does your child have any current medical conditions?
* must provide value

- ◯ Yes
- ◯ No

reset

Please list your child's current medical condition(s): [ ]

Is your child currently taking any prescription medication?
* must provide value

- ◯ Yes
- ◯ No
- ◯ Prefer not to answer

reset

Please list the name of the prescription medication(s): [ ]

Baseline Survey-household

Since January 2020, did your child leave the home to go to:
* must provide value

- ☐ Work *(choose all that apply)*
  - ☐ Healthcare worker
  - ☐ Essential worker (e.g. postal, grocery, delivery)
  - ☐ Personal service provider (e.g. hairdresser/barber, esthetician)
  - ☐ Teacher, Principal, School counselor etc.
  - ☐ Other, specify:
- ☐ School *(choose level of schooling)*
  - ○ Preschool
  - ○ Elementary/Primary
  - ○ Secondary/High school
  - ○ Post secondary (college, university or technical institute)
- ☐ Daycare
- ☐ Other, specify: ______
- ☐ None of the above
- ☐ Prefer not to answer

In the last 6 months, on average, how many times per week does your child use the following transportation?

	never	less than 1/week	1-2 times/week	3-4 times/week	5-6 times/week	daily
**Public transportation** * must provide value	○	○	○	○	○	○ reset
**Another's private vehicle (such as taxi, car pooling, ride share)** * must provide value	○	○	○	○	○	○ reset

COVID-19 EXPOSURE INFORMATION

Since January 2020, has your child travelled outside of BC?

(tick all that apply)
* must provide value

- ☐ Yes, within Canada
- ☐ Yes, outside Canada
- ☐ No
- ☐ Prefer not to answer

Which province did your child travel to?
* must provide value

- ☐ Alberta
- ☐ Manitoba
- ☐ New Brunswick
- ☐ Newfoundland and Labrador
- ☐ Northwest Territories
- ☐ Nova Scotia
- ☐ Nunavut
- ☐ Ontario
- ☐ Prince Edward Island
- ☐ Quebec
- ☐ Saskatchewan
- ☐ Yukon
- ☐ Prefer not to answer

How many total weeks did your child spend outside of BC but within Canada?
* must provide value
______

When did your child last return to BC from within Canada?
* must provide value
______ Today D-M-Y

What country/countries did your child travel to?
* must provide value
______

How many total weeks did your child spend outside of Canada?
* mus: provide value
______

When did your child last return to BC from outside Canada?
* must provide value
______ Today D-M-Y

Baseline Survey-household

Has your child ever had any exposure to a person who tested positive for COVID-19?
* must provide value

- ◯ Yes
- ◯ No
- ◯ I don't know
- ◯ Prefer not to answe

reset

How many people has your child been exposed to?
* must provide value

____

Exposure #1

Please identify where the exposure occurred: (check all that apply)

- ☐ Household member
- ☐ Non-household close contact (friend/other family)
- ☐ Health care setting (e.g. Dr. office, physiotherapis/chiropractor office, hospital)
- ☐ School, college, university
- ☐ Personal service provider (e.g. hair salon/barber, esthetician)
- ☐ Workplace
- ☐ Travel, where:
- ☐ Other, specify: ____________
- ☐ Prefer not to answer

When did this exposure occur?

Month: [ ] **Year:** [ ]

At the time of exposure, do you think the person was still infectious (this is typically within the first 14 days of their illness)?

- ◯ Yes
- ◯ No
- ◯ I don't know
- ◯ Prefer not to answer

Exposure #2

Please identify where the exposure occurred: (check all that apply)

- ☐ Household member
- ☐ Non-household close contact (friend/other family)
- ☐ Health care setting (e.g. Dr. office, physiotherapis/chiropractor office, hospital)
- ☐ School, college, university
- ☐ Personal service provider (e.g. hair salon/barber, esthetician)
- ☐ Workplace
- ☐ Travel, where:
- ☐ Other, specify: ____________
- ☐ Prefer not to answer

When did this exposure occur?

Month: [ ] **Year:** [ ]

At the time of exposure, do you think the person was still infectious (this is typically within the first 14 days of their illness)?

- ◯ Yes
- ◯ No
- ◯ I don't know
- ◯ Prefer not to answer

Exposure #3

Please identify where the exposure occurred: (check all that apply)

- ☐ Household member
- ☐ Non-household close contact (friend/other family)
- ☐ Health care setting (e.g. Dr. office, physiotherapis/chiropractor office, hospital)
- ☐ School, college, university
- ☐ Personal service provider (e.g. hair salon/barber, esthetician)
- ☐ Workplace
- ☐ Travel, where:
- ☐ Other, specify: ____________
- ☐ Prefer not to answer

When did this exposure occur?

Month: [ ] **Year:** [ ]

At the time of exposure, do you think the person was still infectious (this is typically within the first 14 days of their illness)?

- ◯ Yes
- ◯ No
- ◯ I don't know
- ◯ Prefer not to answer

Is your child currently in isolation?

- ◯ Yes
- ◯ No

reset

When your child was in indoor public spaces (e.g. shopping, dining) how frequently did your child:

	Never	Rarely	Some of the time	Most of the time	Every time
**Wash hands or use hand sanitizer** * must provide value	◯	◯	◯	◯	◯
**Maintain a distance of 2 meters from members of the public** * must provide value	◯	◯	◯	◯	◯
**Wear a mask or other face covering** * must provide value	◯	◯	◯	◯	◯

reset

reset

Baseline Survey-household

HOUSEHOLD DETAILS

Other than your child, how many people live in your home since January 2020?
* must provide value

Please complete the following table(s) for each household member:

Household member #1

**Age in years** (enter as "0" for children less than 1yr old)	
**In the last 6 months, did this household member leave the home to go to:** (tick all that apply)	☐ Work *(choose all jobs in the last 6 months)*<br>☐ Healthcare worker<br>☐ Essential worker (e.g. postal, grocery, delivery)<br>☐ Personal service provider (e.g. hairdresser/barber, esthetician)<br>☐ Teacher, Principal, School counsellor etc.<br>☐ Other, specify:<br>☐ School *(choose level of schooling)*<br>○ Preschool<br>○ Elementary/Primary<br>○ Secondary/High school<br>○ Post secondary (college, university or technical institute)<br>reset<br>☐ Daycare<br>☐ Other, specify:<br>☐ No, none of the above

Household member #2

**Age in years** (enter as "0" for children less than 1yr old)	
**In the last 6 months, did this household member leave the home to go to:** (tick all that apply)	☐ Work *(choose all jobs in the last 6 months)*<br>☐ School *(choose level of schooling)*<br>☐ Daycare<br>☐ Other, specify:<br>☐ No, none of the above

⋮

Household member #9

**Age in years** (enter as "0" for children less than 1yr old)	
**In the last 6 months, did this household member leave the home to go to:** (tick all that apply)	☐ Work *(choose all jobs in the last 6 months)*<br>☐ School *(choose level of schooling)*<br>☐ Daycare<br>☐ Other, specify:<br>☐ No, none of the above

Baseline Survey-household

Since January 2020, has your child been sick?
* must provide value

- ○ Yes
- ○ No
- ○ Prefer not to answer

reset

How many times has your child been sick?
* must provide value

[ ]

Illness #1	Illness #2	Illness #3
**When was this illness? (Month/year)**		
[▾] [▾]	[▾] [▾]	[▾] [▾]
**Which of the following symptoms did your child experience during this illness? (tick all that apply)**		
☐ Fever >38 C<br>☐ Chills<br>☐ Cough<br>☐ Shortness of breath<br>☐ Alteration to taste<br>☐ Alteration to smell<br>☐ Nausea<br>☐ Vomiting<br>☐ Diarrhea<br>☐ None of the above<br>☐ Prefer not to answer	☐ Fever >38?C<br>☐ Chills<br>☐ Cough<br>☐ Shortness of breath<br>☐ Alteration to taste<br>☐ Alteration to smell<br>☐ Nausea<br>☐ Vomiting<br>☐ Diarrhea<br>☐ None of the above<br>☐ Prefer not to answer	☐ Fever >38?C<br>☐ Chills<br>☐ Cough<br>☐ Shortness of breath<br>☐ Alteration to taste<br>☐ Alteration to smell<br>☐ Nausea<br>☐ Vomiting<br>☐ Diarrhea<br>☐ None of the above<br>☐ Prefer not to answer
**How long did this illness last?**		
[ ]<br>○ Days<br>○ Weeks<br>reset	[ ]<br>○ Days<br>○ Weeks<br>reset	[ ]<br>○ Days<br>○ Weeks<br>reset
**Did this illness cause your child to miss work or school/daycare?**		
○ Yes<br>○ No<br>reset	○ Yes<br>○ No<br>reset	○ Yes<br>○ No<br>reset
**Did your child need to seek medical attention?**		
○ Yes<br>○ No<br>reset	○ Yes<br>○ No<br>reset	○ Yes<br>○ No<br>reset

You mentioned above that your child sought medical attention, did your child require hospitalization?
* must provide value

- ○ Yes
- ○ No
- ○ Prefer not to answer

reset

Did your child require admission to ICU?
* must provide value

- ○ Yes
- ○ No
- ○ Prefer not to answer

reset

Is your child still sick?
* must provide value

- ○ Yes
- ○ No
- ○ Prefer not to answer

reset

Has your child ever been tested for COVID-19?
* must provide value

- ○ Yes
- ○ No
- ○ Prefer not to answer

reset

When was your child last tested?
* must provide value

mo: [▾] yr: [▾]

What was the result?
* must provide value

- ○ Positive
- ○ Negative
- ○ Indeterminate/Inconclusive
- ○ I don't know
- ○ Prefer not to answer

reset

Baseline Survey-household

Has your child been vaccinated against COVID-19?
* must provide value

- ◯ Yes
- ◯ No

reset

How many doses of the COVID-19 vaccine has your child received so far?
* must provide value

- ◯ One dose
- ◯ Two dose
- ◯ More than two dose

Vaccination date of first dose:

Day	Month	Year

* must provide value

Which vaccine did your child receive?
* must provide value

- ◯ Pfizer and BioNTech mRNA vaccine
- ◯ Moderna mRNA vaccine
- ◯ AstraZeneca Oxford vaccine
- ◯ Janssen (Johnson & Johnson) vaccine
- ◯ Medicago vaccine
- ◯ Novavax vaccine
- ◯ Sanofi/GSK vaccine
- ◯ Other: ______
- ◯ Don't know

Vaccination date of second dose:

Day	Month	Year

* must provide value

Which vaccine did your child receive?
* must provide value

- ◯ Pfizer and BioNTech mRNA vaccine
- ◯ Moderna mRNA vaccine
- ◯ AstraZeneca Oxford vaccine
- ◯ Janssen (Johnson & Johnson) vaccine
- ◯ Medicago vaccine
- ◯ Novavax vaccine
- ◯ Sanofi/GSK vaccine
- ◯ Other: ______
- ◯ Don't know

reset

Vaccination date of additional dose:

Day	Month	Year

* must provide value

Which vaccine did your child receive?
* must provide value

- ◯ Pfizer and BioNTech mRNA vaccine
- ◯ Moderna mRNA vaccine
- ◯ AstraZeneca Oxford vaccine
- ◯ Janssen (Johnson & Johnson) vaccine
- ◯ Medicago vaccine
- ◯ Novavax vaccine
- ◯ Sanofi/GSK vaccine
- ◯ Other: ______
- ◯ Don't know

Please make sure you click the "submit" button to complete your survey. Your survey responses will not be registered if you do not click "submit".

Follow-up Survey

Data Access Group: **[No Assignment]** ?

Invitation status: Survey options

Editing existing Record ID: 3.

Event: **Visit 3 (Arm 1: Arm 1)**

Record ID: 3

BACKGROUND INFORMATION Please answer the following questions about yourself.

PARENTS: if you are answering this survey on behalf of your child, please answer the questions in relation to your child.

On your last survey completed on _____ 04/28/2022 you indicated that you (your child) had the following medical conditions:

_____

Has there been any new or changes to your (your child's) medical conditions?
* must provide value

(x) Yes
( ) No

reset

Please list your (your child's) new or changed medical condition(s): [ ]

On your last survey completed on _____ 04/28/2022, you indicated that you (your child) were taking following prescription medications:

_____

Has there been any new or changes to your (your child's) prescription medications?
* must provide value

(x) Yes
( ) No
( ) Prefer not to answer

reset

Please list the name of the prescription medication(s): [ ]

On your last survey completed on _____ 04/28/2022 you indicated ("checked") that you (your child) left home for the following reasons:

Work: Unchecked - _____

School: Unchecked - _____

Daycare: Unchecked

Other: Unchecked - _____

None: Unchecked

Decline: Unchecked

Follow-up survey continued

Has your (your child's) job or type of schooling changed?
* must provide value

- (●) Yes
- ( ) No
- ( ) Prefer not to answer

reset

Did you (your child) leave the home to go to:
* must provide value

- [x] Work *(choose all that apply)*
  - [ ] Healthcare worker
  - [ ] Essential worker (e.g. postal, grocery, delivery)
  - [ ] Personal service provider (e.g. hairdresser/barber, esthetician)
  - [ ] Teacher, Principal, School counselor etc.
  - [ ] Other, specify:
  - [ ] Prefer not to answer
- [x] School *(choose level of schooling)*
  - ( ) Preschool
  - ( ) Elementary/Primary
  - ( ) Secondary/High school
  - ( ) Post secondary (college, university or technical institute)
  - ( ) Prefer not to answer

  reset
- [ ] Daycare
- [ ] Other, specify: __________

  50 characters remaining
- [ ] None of the above
- [ ] Prefer not to answer

Since your last survey completed on _____ 04/28/2022:

On average, how many times per week do you (your child) use the following transportation?

	never	less than 1/week	1-2 times/week	3-4 times/week	5-6 times/week	daily
**Public transportation** * must provide value	( )	( )	( )	( )	( )	( )
**Another's private vehicle (such as taxi, car pooling, ride share)** * must provide value	( )	( )	( )	( )	( )	( )

reset

Since your last survey completed on _____ 04/28/2022:

Have you (your child) had any exposure to a person who tested positive for COVID-19?
* must provide value

- (●) Yes
- ( ) No
- ( ) I don't know
- ( ) Prefer not to answer

reset

How many people have you (your child) been exposed to?
* must provide value

- ( ) Less than 5 people
- ( ) 5-10 people
- ( ) 10-20 people
- ( ) Greater than 20 people

reset

Follow-up survey continued

Please identify where the most recent exposure occurred: (check all that apply)

- ☐ Household member
- ☐ Non-household close contact (friend/other family)
- ☐ Health care setting (e.g. Dr. office, physiotherapis/chiropractor office, hospital)
- ☐ School, college, university
- ☐ Daycare
- ☐ Personal service provider (e.g. hair salon/barber, esthetician)
- ☐ Workplace
- ☐ Travel, where:
- ☐ Other, specify:
- ☐ Prefer not to answer

When did this exposure occur?

Month:

Year:

At the time of exposure, do you think the person was still infectious (this is typically within the first 14 days of their illness)?

- ○ Yes
- ○ No
- ○ I don't know
- ○ Prefer not to answer

reset

HOUSEHOLD DETAILS

Other than yourself (your child), how many people currently live in your home?

If you are a parent filling this out for your child, please include yourself in the following table as a household member. If you are a student currently living in student residence, please complete the following table for each roommate you have."

* must provide value

2

Household member #1

**Age in years** **(enter as "0" for children less than 1yr old)**	

Household member #2

**Age in years** **(enter as "0" for children less than 1yr old)**	

Follow-up survey continued

Since your last survey completed on _____ 04/28/2022:

Have you (your child) been sick that included one or more of the following symptoms:

- Fever >38°C
- Chills
- Cough
- Shortness of breath
- Alteration to taste
- Alteration to smell
- Nausea
- Vomiting
- Diarrhea

* must provide value

◉ Yes
◯ No
◯ Prefer not to answer

reset

How many times have you (your child) been sick?

* must provide value

4

Please record details on the three illnesses of most concern

Illness #1	Illness #2	Illness #3
When was this illness? (Month/year)		
mo: yr:	mo: yr:	mo: yr:
Did this illness cause you (your child) to miss work or school/daycare?		
◯ Yes ◯ No ◯ Prefer not to answer reset	◯ Yes ◯ No ◯ Prefer not to answer reset	◯ Yes ◯ No ◯ Prefer not to answer reset
Did you (your child) need to seek medical attention?		
◯ Yes ◯ No ◯ Prefer not to answer reset	◯ Yes ◯ No ◯ Prefer not to answer reset	◯ Yes ◯ No ◯ Prefer not to answer reset

Follow-up survey continued

Have you (your child) ever tested positive for COVID-19?
* must provide value

- (●) Yes
- ( ) No
- ( ) Prefer not to answer

reset

How many times have you (your child) tested positive for COVID-19?
* must provide value

10

Please enter your (your child's) POSITIVE tests:

	Month of Test	Year of Test	Test Type
Positive COVID-19 Test #1:			
Positive COVID-19 Test #2:			
Positive COVID-19 Test #3:			
Positive COVID-19 Test #4:			
Positive COVID-19 Test #5:			
Positive COVID-19 Test #6:			
Positive COVID-19 Test #7:			
Positive COVID-19 Test #8:			
Positive COVID-19 Test #9:			
Positive COVID-19 Test #10:			

Follow-up survey continued

COVID-19 Vaccinations

On previous surveys, you indicated that you (your child) had received the following Dose 1 of the COVID-19 vaccines:

07-04-2022, Pfizer and BioNTech mRNA vaccine

Is this dose 1 information correct?

If the field is empty, and you have recived a dose 1 vaccine, please select "No"

* must provide value

- ○ Yes
- ◉ No

reset

	Vaccination Date	Type of Vaccination
**Please enter new or updated Dose 1 information:**	[ ] D-M-Y	○ Pfizer and BioNTech mRNA vaccine<br>○ Moderna mRNA vaccine<br>○ AstraZeneca Oxford vaccine<br>○ Janssen (Johnson & Johnson) vaccine<br>○ Medicago vaccine<br>○ Novavax vaccine<br>○ Sanofi/GSK vaccine<br>○ Other:<br>○ Don't know<br>reset

On previous surveys, you indicated that you (your child) had received the following Dose 2 of the COVID-19 vaccines:

_____, _____

Is this dose 2 information correct?

If the field is empty, and you have recived a dose 2 vaccine, please select "No"

* must provide value

- ○ Yes
- ◉ No

reset

	Vaccination Date	Type of Vaccination
**Please enter new or updated Dose 2 information:**	[ ] D-M-Y	○ Pfizer and BioNTech mRNA vaccine<br>○ Moderna mRNA vaccine<br>○ AstraZeneca Oxford vaccine<br>○ Janssen (Johnson & Johnson) vaccine<br>○ Medicago vaccine<br>○ Novavax vaccine<br>○ Sanofi/GSK vaccine<br>○ Other:<br>○ Don't know<br>reset

On previous surveys, you indicated that you (your child) had received the following Dose 3 of the COVID-19 vaccines:

_____, _____

Is this dose 3 information correct?

If the field is empty, and you have recived a dose 3 vaccine, please select "No"

* must provide value

- ○ Yes
- ◉ No

reset

	Vaccination Date	Type of Vaccination
**Please enter new or updated Dose 3 information:**	D-M-Y	○ Pfizer and BioNTech mRNA vaccine<br>○ Moderna mRNA vaccine<br>○ AstraZeneca Oxford vaccine<br>○ Janssen (Johnson & Johnson) vaccine<br>○ Medicago vaccine<br>○ Novavax vaccine<br>○ Sanofi/GSK vaccine<br>○ Other:<br>○ Don't know<br>reset

On previous surveys, you indicated that you (your child) had received the following Dose 4 of the COVID-19 vaccines:

_____, _____

Is this dose 4 information correct?

If the field is empty, and you have recived a dose 4 vaccine, please select "No"

* must provide value

○ Yes
◉ No

reset

	Vaccination Date	Type of Vaccination
**Please enter new or updated Dose 4 information:**	D-M-Y	○ Pfizer and BioNTech mRNA vaccine<br>○ Moderna mRNA vaccine<br>○ AstraZeneca Oxford vaccine<br>○ Janssen (Johnson & Johnson) vaccine<br>○ Medicago vaccine<br>○ Novavax vaccine<br>○ Sanofi/GSK vaccine<br>○ Other:<br>○ Don't know<br>reset

On previous surveys, you indicated that you (your child) had received the following Dose 5 of the COVID-19 vaccines:

_____, _____

Is this dose 5 information correct?

If the field is empty, and you have recived a dose 5 vaccine, please select "No"

* must provide value

○ Yes
◉ No

reset

	Vaccination Date	Type of Vaccination
**Please enter new or updated Dose 5 information:**	D-M-Y	○ Pfizer and BioNTech mRNA vaccine<br>○ Moderna mRNA vaccine<br>○ AstraZeneca Oxford vaccine<br>○ Janssen (Johnson & Johnson) vaccine<br>○ Medicago vaccine<br>○ Novavax vaccine<br>○ Sanofi/GSK vaccine<br>○ Other:<br>○ Don't know<br>reset
