# Supplementary figures and images for: Factors associated with SARS-CoV-2 infection in unvaccinated children and young adults
Source: BMC Infect Dis. 2024 Jan 15;24:91. doi: 10.1186/s12879-023-08950-1 (PMC10790408; doi:10.1186/s12879-023-08950-1)

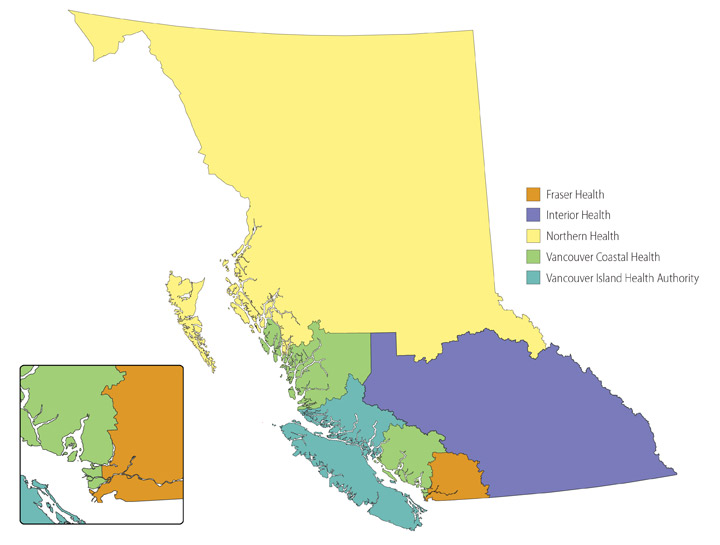

Supplement: Supplementary file 4 — Supplementary Material 4: Supplementary Fig.3: Map of health authorities in British Columbia, Canada. [file 12879_2023_8950_MOESM4_ESM.jpg]
